# Vulnerabilities associated with violence against women before entering the prison system

**DOI:** 10.1590/1980-220X-REEUSP-2022-0167en

**Published:** 2022-10-03

**Authors:** Tyane Mayara Ferreira de Oliveira, Hellen Lívia Oliveira Catunda Ferreira, Vívien Cunha Alves de Freitas, Fabiane da Silva Severino Lima, Flávia Ximenes Vasconcelos, Nicolau da Costa, Ana Karina Bezerra Pinheiro

**Affiliations:** 1 Universidade Federal do Ceará, Departamento de Enfermagem, Programa de Pós-Graduação em Enfermagem. Fortaleza, CE, Brazil.

**Keywords:** Health Vulnerability, Violence Against Women, Prisons, Prisoners, Vulnerabilidad en Salud, Violencia contra la Mujer, Prisiones, Prisioneros, Vulnerabilidade em Saúde, Violência contra a Mulher, Prisões, Prisioneiros

## Abstract

**Objective:**

to analyze the individual and social vulnerabilities of women deprived of their liberty for violence suffered before entering the prison system.

**Method:**

an analytical crosssectional study, carried out with 272 inmates of a female prison unit, in the Metropolitan Region of Fortaleza, Ceará. We applied two instruments: a form to analyze sociodemographic information and the violence suffered prior to entering the prison and the Alcohol, Smoking and Substance Involvement Screening Test (ASSIST), which analyzed the history of psychoactive substance use.

**Results:**

44.5% of women suffered violence. Most of the total sample was between 18 and 29 years old, with children, low education and income, early onset of sexual life and history of illicit drug use. Age between 18 and 29 years proved to be a protective factor against violence (OR = 0.632). Cocaine and crack use (p =0.002), amphetamines and ecstasy (p =0.018) increase the chance of violence by 2.2 to 3.3 times.

**Conclusion:**

aspects of the individual and social dimensions of vulnerability are associated with the occurrence of violence in women in the female prison system. Effective strategies need to be designed based on vulnerabilities to prevent violence against women.

## RESUMO

**Objetivo:**

analisar as vulnerabilidades individuais e sociais de mulheres privadas de liberdade para violência sofrida antes da entrada no sistema prisional.

**Método:**

estudo transversal analítico, realizado com 272 internas de uma unidade prisional feminina, na Região Metropolitana de Fortaleza, Ceará. Foram aplicados dois instrumentos: formulário para análise de informações sociodemográficas e da violência sofrida previamente à entrada no presídio e o Alcohol, Smoking and Substance Involvement Screening Test (ASSIST), que analisou o histórico do uso de substâncias psicoativas.

**Resultados:**

44,5% das mulheres sofreram violência. A maior parte da amostra total apresentava idade entre 18 e 29 anos, com filhos, baixa escolaridade e renda, início precoce da vida sexual e histórico de uso de drogas ilícitas. A idade entre 18 e 29 anos mostrou ser fator protetor da violência (OR = 0,632). Uso de cocaína e crack (p = 0,002), anfetaminas e êxtase (p = 0,018) aumenta a chance de violência de 2,2 a 3,3 vezes.

**Conclusão:**

aspectos das dimensões individuais e sociais da vulnerabilidade estão associadas à ocorrência de violência em mulheres internas do sistema prisional feminino. Estratégias efetivas necessitam ser traçadas com base nas vulnerabilidades, para prevenir violência contra a mulher.

## RESUMEN

**Objetivo:**

analizar las vulnerabilidades individuales y sociales de las mujeres privadas de libertad por la violencia sufrida antes de ingresar al sistema penitenciario.

**Método:**

estudio transversal analítico, realizado con 272 internas de una unidad penitenciaria femenina, en la Región Metropolitana de Fortaleza, Ceará. Se aplicaron dos instrumentos: un formulario para analizar la información sociodemográfica y la violencia sufrida antes del ingreso al penal y el Alcohol, Smoking and Substance Involvement Screening Test (ASSIST), que analizó el historial de uso de sustancias psicoactivas.

**Resultados:**

El 44,5% de las mujeres sufrieron violencia. La mayor parte de la muestra total tenía entre 18 y 29 años, con hijos, baja escolaridad e ingresos económicos, inicio temprano de la vida sexual y antecedentes de consumo de drogas ilícitas. La edad entre 18 y 29 años resultó ser un factor protector contra la violencia (OR =0,632). El consumo de cocaína y crack (p =0,002), anfetaminas y éxtasis (p =0,018) aumenta la probabilidad de violencia entre 2,2 y 3,3 veces.

**Conclusión:**

aspectos de las dimensiones individual y social de la vulnerabilidad están asociados a la ocurrencia de violencia en mujeres en el sistema penitenciario femenino. Es necesario diseñar estrategias efectivas basadas en las vulnerabilidades para prevenir la violencia contra las mujeres.

## INTRODUCTION

Violence against women is a critical public health concern and determinant in the health-disease process. It is a persistent global phenomenon in today’s societies, which manifests itself in different forms (physical, sexual and psychological) and at different levels of severity. It is an expression of socially constructed gender inequality, in which power relations, resulting from victim-aggressor duality, weaken women’s autonomy^(1,2)^.

Lifelong violence, especially when sexual, can lead to behaviors and health conditions, which result in triggering acts in prison detention. It is estimated that 56 to 82% of incarcerated women suffered sexual abuse at some point in their lives, being associated with the development of mental illness and substance abuse^(3)^.

Cases of violence against women are particularly high in the prison population. In São Paulo, Brazil alone, 40% of them have suffered psychological violence and 31% have experienced some type of physical/sexual violence before entering the prison system. More robust data on violence prior to entering the prison system in the lives of these women are still rare or nonexistent^(4)^.

Acts of violence against women deprived of liberty can be influenced by a set of aspects related to the individual and the community, called vulnerabilities, which can be individual, social or programmatic^(5)^.

Individual vulnerability concerns the degree and quality of information that individuals have about the health problem, their ability to incorporate it into their daily repertoire of concerns, and their interest in effective possibilities for transforming these concerns into health-protective practices. Social vulnerability involves aspects that enable access to information, spending on social and health services, and it includes the life cycle, social mobility, social identity and the power to incorporate them into practical changes. Programmatic vulnerability is related to the quality of commitment, resources, management and monitoring of national, regional or local prevention programs and health care^(5)^.

Thus, it is understood that knowing the violence suffered by a woman before entering the prison system, the individual and social vulnerabilities related to it can be a relevant factor to plan and develop effective public policy actions for the prevention of both violence itself and acts linked to the prison process, contributing to the mitigation of vulnerabilities. Thus, this study aims to analyze the individual and social vulnerabilities of women in deprivation of liberty for violence suffered before entering the prison system.

## METHOD

### Study Design

This is an analytical and quantitative cross-sectional study, which involves a more in-depth assessment of the data collected in an observational or experimental study, in an attempt to explain the context of a phenomenon, i.e., its relationship between cause and effect^(6)^. To perform the analysis, the international protocol Strengthening the Reporting of Observational Studies in Epidemiology (STROBE) was used, validated according to the observational research design.

### Setting

The research was conducted with inmates of a female prison unit, located in the metropolitan region of Fortaleza, Ceará. Inaugurated in 1974, the unit underwent modifications, and in 2000, the new and current building was launched in the city of Aquiraz, with capacity for 380 inmates.

### Sample Definition

For the sample calculation, non-probabilistic convenience sampling was used, due to the difficulty of preparing probabilistic and representative samples of the prison unit. Thus, the final sample corresponded to 272 inmates, who met the criteria and accepted the invitation to participate in the research, carried out at the time of collection, after explaining the objectives and reading the Informed Consent Term (ICF).

### Selection Criteria

All female inmates, regardless of the history of violence, who agreed to participate in the study were included. Those that, for some reason related to the institutional routine, were not available at the time of data collection were excluded.

### Data Collection

Data collection occurred in July 2020, together with nurses working in the prison unit. It is noteworthy that, given the impossibility of moving the inmates to another space, the meetings for data collection took place at the door of the cells of previously selected wards, following guidelines from the coordination, with an average duration of 25 minutes each.

As a way to ensure privacy, ensure comfort in the face of possibilities and meet the routine applied in the unit, data collection took place as follows: while one participant was collected by the researcher or one of the nurses, the others stayed away (creating a space between the inmate and the other people in the cell), sitting with their backs and in silence, waiting for the end of the application of the instrument. The unit nurses, in turn, were previously selected by the Department of Penitentiary Administration’s (DPA) Health Coordinator, and trained prior to data collection, with the explanation of the instrument’s questions, as well as its completion, aiming to clarify doubts and avoid bias.

Two instruments were used for collection. The first was a structured form with closed-ended questions, previously validated^(7)^, which analyzed: sociodemographic information (gender identity, age, race, marital status, education, occupation, children, income, religion, feeling welcomed by the region and coitarche); violence suffered prior to entering the prison; and drug use prior to entering the prison during sexual intercourse with a fixed and/or casual partner. Although the types of vulnerability are considered inseparable, programmatic vulnerability was not addressed in this study, due to the nature of the collection instrument and the difficulty of verifying data related to this domain with the target population.

The second instrument was the Alcohol, Smoking and Substance Involvement Screening Test (ASSIST), also validated for Brazil^(8)^, used to collect data on the history of psychoactive substance use (tobacco, alcohol, marijuana, cocaine, stimulants, sedatives, inhalants, hallucinogens and opiates) prior to entering the prison system.

### Data Analysis and Treatment

The association of individual and social vulnerabilities was made^(5)^ of women in deprivation of liberty with violence that occurred before entering the prison system and general violence (question regarding whether the woman suffered any type of violence among the possible types) and with each type of violence suffered (physical, psychological, moral, sexual and patrimonial). The definition of types of violence was explained to participants at the time of collection, based on the document available in the “instruction for reporting domestic, sexual and other violence”, prepared by the Ministry of Health. The individual vulnerabilities assessed were gender identity, race, age, education, marital status and children. Social vulnerabilities were employment, income, religion, acceptance in their religion, age of coitarche and illicit substance use.

The final version of the database was transported from Microsoft Excel® to Stata, version 16.0. Data processing and analysis procedures started with the calculation of gross distributions and percentages for sociodemographic determinants, violence suffered and individual and social vulnerabilities.

Fisher’s exact or chi-square tests were applied to identify an association between individual/social vulnerabilities regarding substance use and the occurrence of some type of violence, so the set of vulnerabilities was analyzed separately. Subsequently, logistic regression techniques were adopted to identify the risk associated with the occurrence of some type of violence, with the presentation of the respective 95% confidence intervals.

Using the stepwise method, it was followed to elaborate a final reduced model, in order to obtain the best model adjusted for the outcome. For this, the variables with a p-value lower than 0.20 were managed in the bivariate analyses. The assessment of the final model’s fit occurred through the Hosmer and Lemeshow, Omnibus and Nagelkerke’s R^2^ tests. The results were presented in tables. The significance level used throughout the analysis was 5%.

### Ethical Aspects

We respected the ethical and legal principles in force in Brazil, according to the Brazilian National Health Council, established in Resolution 466/2012. A request for authorization for the research was requested from the Penitentiary Administration’s Special Coordination, which issued a favorable opinion for its performance. Moreover, the project was submitted for assessment by the Research Ethics Committee of the *Universidade Federal do Ceará*, with approval on March 17, 2020, through Opinion 3,921,161. Given the scenario of the COVID-19 pandemic, during data collection, the health protocols in force at the time were complied with, such as the correct use of the necessary Personal Protective Equipment (PPE), distancing and hand hygiene. To participate in the research, participants were informed about the research objectives and signed the ICF, in two copies, one being delivered to the participant and the other staying with the researcher.

## RESULTS

Regarding gender identity, the majority declared themselves to be women (88.29%), aged between 18 and 29 years (64.71%), brown (79.78%), with children (75.53%) and without a partner (56.25%). They had low education, with up to nine years of education (59.93%) and reported being economically active (52.57%), but had no income in the last 30 days (54.41%). Most (83.46%) had religion and reported feeling welcomed by it (79.04%), and 82.35% started sexual life early (before 15 years). About drug use during sexual intercourse, 44.85% reported that they never used it with their fixed partners, 22.43% always used it and 19.12% used it sometimes. Regarding drug use during sexual intercourse with a casual partner, the majority (80.88%) reported never having used them.

Also, it was found that about 44.5% of women deprived of liberty reported having suffered some type of violence before entering the prison. The main types of violence mentioned were physical (26.10%), psychological (23.53%) and sexual (16.18%).

Thus, it was identified (Table 1) that, among the individual dimensions, there was a higher prevalence of reports of violence among participants who identified themselves as women (44.58%), aged 30 years or older (50%), of yellow race (62.5%), without a partner (45.1%), with low education (47.8%) and who had children (45.5%). In relation to social aspects, the highest prevalence was among participants who had an occupation (44.8%), those whose income was from one to two minimum wages (66.7%), with a religion (45.8%), but who did not feel welcomed by it (58.3%), sexual life started up to the age of 15 (45.5%), who most often used drugs in relationships with a fixed partner before being arrested (53.8%) and who never used drugs during sexual intercourse with a casual partner (45.9%). However, no association was statistically significant.

**Table 1:** Association between the individual and social dimensions of vulnerability and the report of some type of violence. N = 272 − Fortaleza, CE, Brazil, 2021.

Sociodemographic characterization	Report of some violence				p-value	OR^¶^	95%CI
	Yes		No				
	N	%	N	%			
**Gender identity**					0.929^‡^		
Women	107	44.58	133	55.42		1.00	–
Not woman	14	43.75	18	56.25		0.966	0.459; 2.033
**Age group**					0.176^‡^		
18 to 29 years	73	41.48	103	58.52		0.708	0.429; 1.168
30 years and older	48	50.00	48	50.00		1.00	–
**Skin color**					0.669‡		
White	14	50.00	14	50.00		1.00	–
Black	8	42.11	11	57.89		0.727	0.224; 2.352
Yellow	5	62.50	3	37.50		1.666	0.332; 8.352
Brown	94	43.32	123	56.68		0.764	0.347; 1.680
**Marital status**					0.296^†^		
Does not know	0	0	3	100.00		⋆	⋆
Without partner	69	45.10	84	54.90		1.01	0.622; 1.642
With partner	52	44.83	64	55.17		1.00	–
**Education**					0.172‡		
Up to 9 years (low education)	78	47.85	85	52.15		1.408	0.862; 2.303
10 years or more (high education)	43	39.45	66	60.55		1.00	–
**Occupation**					0.925‡		
Inactive	57	44.19	72	55.81		1.00	–
Active	64	44.76	79	55.24		0.977	0.605; 1.577
**Children**					0.575^‡^		
Yes	91	45.50	109	54.50		1.168	0.677; 2.015
No	30	41.67	42	58.33		1.00	–
**Money received in the last 30 days**					0.655^†^		
No income	63	42.57	85	57.43		1.00	–
Less than a MW"	16	41.03	23	58.97		0.938	0.458; 1.921
Between one MW" and two MW"	2	66.67	1	33.33		2.698	0.239; 30.420
Does not know the value	40	48.78	42	51.22		1.284	0.747; 2.209
**Religion**					0.322^‡^		
Without religion	17	37.78	28	62.22		1.00	–
With religion	104	45.81	123	54.19		0.718	0.372; 1.384
**Welcomed by religion**					0.409^‡^		
Does not have religion	17	37.78	28	62.22		⋆	⋆
Yes	97	45.12	118	54.88		1.0	–
No	7	58.33	5	41.67		1.703	0.524; 5.535
**Coitarche**					0.624^†^		
Virgin	1	25.00	3	75.00		⋆	⋆
Early sexual life onset (up to 15 years)	102	45.54	122	54.46		1.0	–
Non-early sexual life onset	18	40.91	26	59.09		0.840	0.436; 1.617
**Drug use before being arrested during sex with a fixed partner**					0.374‡		
Always	30	49.18	31	50.82		1.0	–
Most of the time	7	53.85	6	46.15		1.205	0.362; 4.004
Sometimes	17	32.69	35	67.31		0.501	0.233; 1.080
Rarely	12	50.00	12	50.00		1.033	0.401; 2.657
Never	55	45.08	67	54.92		0.848	0.458; 1.570
**Drug use before being arrested during sexual intercourse with casual partner**					0.783^‡^		
Always	7	38.89	11	61.11		1.0	–
Most of the time	1	20.00	4	80.00		0.392	0.036; 4.276
Sometimes	10	41.67	14	58.33		1.122	0.322; 3.908
Rarely	2	40.00	3	60.00		1.047	0.138; 7.934
Never	101	45.91	119	54.09		1.333	0.498; 3.568

* "Minimum wage; ^†^Fisher’s exact test; ^‡^Chi-square test; ^¶^Odds Ratio; ^⋆^Uncalculated values.

As for substance use (Table 2), of the 51 participants, 25% who used tobacco products and 53.03% of those who drank alcohol reported not having suffered any type of violence. Furthermore, there was a statistically significant association (p = 0.034) between marijuana use and reports of some type of violence (48.91% of women), with an increased chance of up to 76% of women who used marijuana to suffer some type of violence (OR = 1,760).

**Table 2:** Association between individual vulnerability regarding substance use and violence report. N = 272 − Fortaleza, CE, Brazil, 2021.

Substances used	Report of some violence				p-value	95%CI^¶^	OR
	Yes		No				
	N	%	N	%			
**Tobacco products**					0.091		
Yes	78	48.75	82	51.25		1.526	0.934; 2.494
No	43	38.39	69	61.61		1.0	–
**Alcoholic beverages**					0.177		
Yes	93	46.97	105	53.03		1.455	0.842; 2.513
No	28	37.84	46	62.16		1.0	–
**Marijuana**					0.034		
Yes	90	48.91	94	51.09		1.760	1.042; 2.973
No	31	35.23	57	64.77		1.0	–
**Cocaine, crack**					<0.001		
Yes	77	54.61	64	45.39		2.378	1.455; 3.888
No	44	33.59	87	66.41		1.0	–
**Amphetamines or ecstasy**					0.003		
Yes	17	73.91	6	26.09		3.950	1.506; 10.360
No	104	41.77	45	58.23		1.0	–
**Inhalants**					0.001		
Yes	36	63.16	21	36.84		2.621	1.433; 4.794
No	85	39.53	130	60.47		1.0	–
**Hypnotics/sedatives**					0.049		
Yes	27	57.45	20	42.55		1.881	0.995; 3.553
No	94	41.78	131	58.22		1.0	–
**Hallucinogens**					0.670		
Yes	7	50.00	7	50.00		1.263	0.430; 3.705
No	114	44.19	144	55.81		1.0	–
**Opioids**					0.876		
Yes	9	42.86	12	57.14		0.930	0.378; 2.287
No	112	44.62	139	55.38		1.0	–
**Others**					0.823		
Yes	2	50.00	2	50.00		1.252	0.173; 9.020
No	119	44.40	149	55.60		1.0	–
**None**					0.249		
Yes	109	45.80	129	54.20		1.549	0.733; 3.273
No	12	35.29	22	64.71		1.0	–

¶ Odds Ratio; ‡Chi-square test.

Regarding cocaine and/or crack use, 54.61% of women reported having suffered some type of violence, with a statistically significant association between cocaine/crack use and violence report (p = 0.001). Women with this profile were 2.4 times more likely to suffer some violence than those who did not use these substances (OR = 2.378). Regarding amphetamines or ecstasy use, 73.91% of women reported having suffered some violence, with a statistically significant association between amphetamines or ecstasy and the report of some violence (p = 0.003). They were 3.9 times more likely to suffer violence than those who did not use this type of drug (OR = 3.950).

As for inhalant use, 63.16% of participants reported having suffered some type of violence, with a statistically significant association between inhalant use, and the report of women who suffered some violence (p = 0.001), presenting 2.6 times more risk of suffering violence in relation to those who did not use such substances (OR = 2.621). Regarding hypnotic/sedative use, 57.45% of interviewees reported having suffered some type of violence, with a statistically significant association between hypnotic/sedative use and the violence report (p = 0.049 − OR = 1.881).

Table 3 below highlights the risk of violence, considering individual and social vulnerabilities. It is verified that the risk of suffering violence in the age group from 18 to 29 years is approximately 40% lower compared to the older age group (OR = 0.632).

**Table 3:** Adjusted logistic regression model for the occurrence of some type of violence, considering individual and social vulnerabilities. N = 272 − Fortaleza, CE, Brazil, 2021.

Variables	S.E.^†^	x^2‡^	gl	p-value	95%CI^¶^	OR
Age	0.168	1.73	1	0.048	0.632	0.375; 0.904
Cocaine, crack	0.572	3.09	1	0.002	2.219	1.338; 3.679
Amphetamines or ecstasy	1.672	2.37	1	0.018	3.312	1.231; 8.910
Constantly	0.090	3.86	1	<0.001	0.486	–
**Model adjustment measures**						
Hosmer and Lemeshow test – p-value				0.491		
Omnibus test – p-value				<0.001		
Nagelkerke R^2^				0.061		

† Standard error; Odds Ratio; ^‡^Chi-square.

In relation to illicit substances, cocaine and crack use is 2.2 times more likely to cause violence to women than not to use (OR = 2.219). Regarding amphetamine or ecstasy use, there is a 3.3 times higher risk of women suffering violence in relation to non-use of these substances (OR = 3.312).

It is also evident that the variables presented, age between 18 and 29 years and illicit substance use, such as cocaine, crack, amphetamines and ecstasy, explain 6.10% of the occurrence of the outcome (R^2^ = 0.061).

## DISCUSSION

The presence of violence was related to dimensions of individual vulnerability, such as the self-identification of being a woman, being 30 years of age or older, being of mixed race, not having a partner, having low education and not having children, despite the association between these variables and the occurrence of violence are not statistically significant.

Social vulnerability dimension aspects were also related to the occurrence of some type of violence. Among them, we mention: having an occupation; have an income of one to two minimum wages; being adept at a religion, despite not feeling welcomed by it; having started sexual life before the age of 15; having the habit of using drugs in relationships with a fixed partner; and having never used it during sexual intercourse with a casual partner. These dimensions were related to the occurrence of violence, although they also do not have a significant statistical association.

From the understanding of the individual and social dimensions of vulnerability addressed(^9^), it was possible to identify which dimensions were related to the occurrence of violence. These dimensions are interrelated and act synergistically and dynamically^(10)^; therefore, they cannot be analyzed in isolation to explain the occurrence of violence itself, but they need to be known so that prevention and health promotion strategies can be thought of in a more realistic, programmatic and ethically oriented way.

Although there is no statistical significance of the association of individual and social dimensions, presented in Table 1, with violence suffered, in general, among the women in the study, the relationship between them is based on the literature. Cases of violence against people deprived of their liberty are related to the living conditions to which women were exposed, individual and social aspects that make people more vulnerable to the occurrence of violence. All this is compounded by persistent inequalities in gender, race, economics and other factors^(11)^.

In an analysis of the individual dimension of gender identity and sexual orientation, it was found in a systematic review that physical violence ranged from 6 to 25%, and sexual violence, from 5.6 to 11.4% among sexual minorities. For transgender people, the prevalence was 11.8 to 68.2% in relation to physical violence, and 7 to 49.1% for sexual violence^(12)^. A second review study showed that the non-white skin color dimension seems to have a positive association with psychological violence, as well as the marital status of not being married seems to be related to a higher prevalence of psychological and physical/sexual violence^(3)^. The low education dimension was associated with the occurrence of violence in a study carried out in Vitória, Espírito Santo, with women in Primary Care, in which the occurrence of psychological (57%), physical (39.3%) and sexual (18) violence was identified. %) among women throughout life^(13)^.

The age of 30 years or more and being brown is a characteristic of most women inserted in the Brazilian prison system^(14)^. The association between skin color and violence is a mark with historical connotations in the country, since, during the Brazilian slavery period, legal and constitutional violence was a seal of absolute power over the black body of men and women. However, sexism and/or racism gains much more substantial impacts, since there is the intersection of two major points of vulnerability to violence, that of being a woman and the color of this woman, because both variables are related to multiple socio-structural violence, due to repression, structural machismo and the culture of the custody system historically experienced by women^(15)^.

The occupation aspect of the social dimension was also assessed in another national study^(16)^, in which it was obtained that most women victims of violence investigated had some formal employment or not. However, other studies suggest that being a housewife or spending more time at home considerably increases a woman’s chances of suffering some type of violence, due to the high rates of domestic violence^(17–19)^. Also, the religion aspect, linked to the social dimension, was more associated with psychological and sexual violence, while the income dimension was related to the occurrence of physical violence. Licit and illicit drug use and a history of aggression, especially in childhood, were also associated with cases of violence^(13)^.

Physical, psychological and sexual violence, identified as more prevalent in the previous life of women deprived of their liberty, were also a reality identified in a study carried out in a prison system in the countryside of the state of São Paulo, where they related such violence to the physical and mental consequences in the future life of women, especially when it occurred before the age of fifteen^(3)^.

Violence against women has consequences for health, which are different if they had occurred with men. It can lead to unwanted pregnancy, sexually transmitted infections, posttraumatic stress disorder, and long-term consequences, which may have a greater impact on the risk of perpetuation of violence with children^(20)^, and trauma, which may permeate future generations.

Situations of stress and conflict, such as in situations of unemployment, social, ecological and natural disasters also seem to be related to the increase in cases of violence against women, especially in home situations. The author brings the reflection that, in times of conflict, as in situations of economic deprivation, violent masculinities are accentuated, due to a correlation between patriarchy and capitalism that creates social norms that dictate that men should be aggressive and economically successful^(21)^. Gender identity issues of violent masculinities need to be better studied and addressed to explain their relationship with the occurrence of violence among women.

The associations were particularly significant between age and violence, revealing a 40% lower risk of women between 18 and 29 years of age suffering violence (Table 3). Another national study verified the prevalence of violence according to the type and age group of the victim, noting that physical (p < 0.001) and sexual (p = 0.002) violence occurred more frequently in women under 18 years of age, while violence psychological (p < 0.001) predominated in women aged between 31 and 40 years. Women over the age of 60 suffered more moral (p < 0.001) and patrimonial violence (p < 0.001)^(22)^.

In univariate analyzes of a Mexican study, intimate partner violence in general (p = 0.034) and specific violence, such as use of severe physical force, forced unwanted sexual behavior and physical isolation (p = 0.015), use of verbal and psychological dominance and social isolation (p = 0.034), in addition to unwanted contact that causes fear and concern about safety (p = 0.003), were significantly associated with age. Women incarcerated under 30 years of age had a lower percentage of reports of violence, in general, by intimate partner, compared to women over 30 years^(11)^, similar to the data found in this study.

Under the analysis of drug use and its association with the occurrence of violence, it was found that marijuana, cocaine and/or crack, amphetamine or ecstasy, inhalant and hypnotic/ sedative use was associated with the occurrence of violence (Table 2). This association between drug use and violence was particularly significant in this study, showing a 2.2-fold higher risk of violence in cocaine and crack users and 3.3 times higher in amphetamine or ecstasy users (Table 3).

A systematic review and meta-analysis of cohort studies found a significant association between violence against women and marijuana use (OR = 2.20 95% CI: 1.52−3.17)^(23)^. Furthermore, a study in an Islamic country identified a higher prevalence of cases of violence when associated with drug use in general^(24)^. A study in Denmark showed that cocaine use can increase the risk of women suffering some kind of violence^(25)^. In a study conducted at the University of Otago Christchurch, New Zealand, regarding amphetamine use and violence against women, a statistically significant association was identified (p < 0.05), with the risk of 1.43 times more likely for women to suffer violence, compared to those who do not use^(26)^. Another study found a significant association between stimulant/inhalant, substance use and some violence (p = 0.036)^(27)^.

Other studies corroborate that female drug users experience high rates of violence, mainly by intimate partners, but also by known and even strangers. Furthermore, experiences of violence, or threat of violence, are associated with risky behaviors related to sexually transmitted diseases, including sharing syringes and reluctance to use health services to reduce harm^(28)^.

Reports of women who inject drugs about experiences of sexual violence throughout their lives, in a study from Puerto Rico, show that unwanted sexual behaviors are the most prevalent, being most commonly forced with threats and physical violence (78%), as well as arguments and continuous pressure (71%), mainly by male aggressors (94%). It is also worth mentioning that more than half of these women stated that they or their aggressors were under the influence of drugs during the time of violence^(29)^.

Bivariate analysis also showed that a greater proportion of women incarcerated in Lima, Peru, who suffered sexual assault, had illicit substance use, in addition to depressive symptoms, HIV or STI infection, and sex work prior to incarceration^(30)^. It was confirmed that illicit drug use can increase the chances of these women being victims of violence or threats of violence, and, ultimately, may increase their general vulnerability, corroborating the present study.

Both in the results presented here and in the literature^(18,29,30)^, the positive relationship between drug use, legal or illicit, with the occurrence of violence against women, was perceived, thus requiring, therefore, that health professionals are prepared to work in the context of primary prevention, with the identification of risk factors associated with the occurrence of violence, assessing women and their context of social life, as in the context of secondary prevention, providing adequate care to victims of violence from physical care to mental health dimensions, avoiding the victim’s feeling of guilt and dependence on substance use, as well as implementing strategies to prevent the recurrence of events^(18)^.

In this context, it is imperative to understand the personal and psychosocial trajectory of incarcerated women and their individual and social dimensions, which are interrelated and that, in some way, influence their current life context.

Thus, this study was limited by the fact that it did not address violence against women deprived of their liberty within the scope of programmatic vulnerability. Furthermore, other variables associated with the occurrence of violence among women also need to be raised and analyzed, since the variables scored here do not explain the entire outcome. These conditions can be considered in the conduct of future research.

## CONCLUSION

The study identified and analyzed the individual and social dimensions of vulnerability, associated with the occurrence of violence in the female prison system. Most of women victims of violence were female, young, with children, without a partner, with low education and income and with early onset of sexual life. Moreover, they had a history of illicit drug use. The main reports of violence were physical, psychological and sexual. Age and illicit drug use were significantly associated with the occurrence of violence. The age between 18 and 29 years was a protective factor. Cocaine, crack, amphetamine and ecstasy use increases the chance of violence by 2.2 to 3.3 times.

From the analysis carried out, such data contribute to the professional practice of nursing, considering that nurses, central agents and leaders of the health team, whether in the community, hospital or prison scope, based on such data, have information that influence the health status of people with some degree of vulnerability, with potential for action and reduction of inequities, enabling health promotion in socially and culturally disadvantaged contexts with society, public security workers and health managers.
